# Registro Multicêntrico de Takotsubo (REMUTA) – Aspectos Clínicos, Desfechos Intra-Hospitalares e Mortalidade a Longo Prazo

**DOI:** 10.36660/abc.20190166

**Published:** 2020-08-19

**Authors:** Gustavo Luiz Gouvêa de Almeida, João Mansur, Denilson Campos de Albuquerque, Sergio Salles Xavier, Álvaro Pontes, Elias Pimentel Gouvêa, Alexandre Bahia Barreiras Martins, Nágela S. V. Nunes, Lilian Vieira Carestiato, João Luiz Fernandes Petriz, Armando Márcio Gonçalves Santos, Bruno Santana Bandeira, Bárbara Elaine de Jesus Abufaiad, Luciana da Camara Pacheco, Maurício Sales de Oliveira, Paulo Eduardo Campana Ribeiro, Pedro Paulo Nogueres Sampaio, Gustavo Salgado Duque, Luiz Felipe Camillis, André Casarsa Marques, Francisco Carlos Lourenço, José Ricardo Palazzo, Cláudio Ramos da Costa, Bibiana Almeida da Silva, Cleverson Neves Zukowski, Romulo Ribeiro Garcia, Fernanda de Carvalho Zonis, Suzana Andressa Morais de Paula, Carolina Gravano Ferraz Ferrari, Bruno Soares da Silva Rangel, Roberto Muniz Ferreira, Bárbara Ferreira da Silva Mendes, Isabela Ribeiro Carvalho de Castro, Leonardo Giglio Gonçalves de Souza, Luiz Henrique dos Santos Araújo, Alexandre Giani

**Affiliations:** 1 Casa de Saúde São José Rio de Janeiro RJ Brasil Casa de Saúde São José,Rio de Janeiro, RJ - Brasil; 2 Hospital Samaritano Rio de Janeiro RJ Brasil Hospital Samaritano,Rio de Janeiro, RJ - Brasil; 3 Universidade do Estado do Rio de Janeiro Rio de Janeiro RJ Brasil Universidade do Estado do Rio de Janeiro,Rio de Janeiro, RJ - Brasil; 4 Instituto D’or de pesquisa e ensino: ID’or/Cardiologia D’or Rio de Janeiro RJ Brasil Instituto D’or de pesquisa e ensino: ID’or/Cardiologia D’or,Rio de Janeiro, RJ - Brasil; 5 Instituto Nacional de Infectologia Evandro Chagas Rio de Janeiro RJ Brasil Instituto Nacional de Infectologia Evandro Chagas – FIOCRUZ,Rio de Janeiro, RJ - Brasil; 6 Universidade Federal do Rio de Janeiro Rio de Janeiro RJ Brasil Universidade Federal do Rio de Janeiro,Rio de Janeiro, RJ - Brasil; 7 Americas Medical City Rio de Janeiro RJ Brasil Americas Medical City,Rio de Janeiro, RJ - Brasil; 8 Complexo Hospitalar de Niterói Niterói RJ Brasil Complexo Hospitalar de Niterói,Niterói, RJ - Brasil; 9 Hospital Universitário Antonio Pedro Niterói RJ Brasil Hospital Universitário Antonio Pedro,Niterói, RJ - Brasil; 10 Clínica São Vicente Rio de Janeiro RJ Brasil Clínica São Vicente,Rio de Janeiro, RJ - Brasil

**Keywords:** Cardiomiopatia Dilatada, Cardiomiopatia de Takotsubo/mortalidade, Insuficiência Cardíaca, Estresse Psicológico, Dor Torácica, Dispneia, Estudo Multicêntrico

## Abstract

**Fundamento:**

A síndrome de takotsubo (takotsubo) é uma forma de cardiomiopatia adquirida. Dados nacionais sobre essa condição são escassos. O Registro REMUTA é o primeiro a incluir dados multicêntricos dessa condição no nosso país.

**Objetivo:**

Descrever as características clínicas, prognóstico, tratamento intra-hospitalar e mortalidade hospitalar e em 1 ano de seguimento.

**Métodos:**

Estudo observacional, retrospectivo, tipo registro. Incluídos pacientes internados com diagnóstico de takotsubo ou que desenvolveram esta condição durante internação por outra causa. Os desfechos avaliados incluíram fator desencadeador, análise dos exames, uso de medicações, complicações e óbito intra-hospitalar e em 1 ano de seguimento. O nível de significância adotado foi de 5%.

**Resultados:**

Foram incluídos 169 pacientes, em 12 centros no Estado do Rio de Janeiro. A idade média foi de 70,9 ± 14,1 anos e 90,5% eram do sexo feminino; 63% dos casos foram de takotsubo primário e 37% secundário. Troponina I foi positiva em 92,5% dos pacientes e a mediana de BNP foi de 395 (176,5; 1725). Supradesnivelamento do segmento ST esteve presente em 28% dos pacientes. A fração de ejeção do ventrículo esquerdo teve mediana de 40 (35; 48)%. Observamos taxa de 25,7% de ventilação mecânica invasiva e 17,4% de choque. Suporte circulatório mecânico foi utilizado em 7,7%. A mortalidade intra-hospitalar foi de 10,6% e a mortalidade ao final de 1 ano foi de 16,5%. Takotsubo secundário e choque cardiogênico foram preditores independentes de mortalidade.

**Conclusão:**

Os resultados do REMUTA mostram que takotsubo não se trata de patologia benigna como se pensava, especialmente no grupo de takotsubo secundário que acarreta elevada taxa de complicações e de mortalidade. (Arq Bras Cardiol. 2020; 115(2):207-216)

## Introdução

A síndrome de takotsubo (takotsubo), também conhecida como cardiomiopatia de takotsubo ou síndrome do coração partido, é uma disfunção regional reversível do ventrículo esquerdo (VE) e/ou direito (VD), na ausência de coronariopatia obstrutiva, sendo provocada, em grande parte dos casos, por situações de estresse agudo. Foi primeiramente descrita por Sato et al., em 1990, no Japão, com uma série de 16 casos que apresentavam características clínicas de síndrome coronariana aguda, porém todas com artérias coronárias angiograficamente normais, tendo histórico de evento estressor que precedia a dor torácica. Seu nome se deve à comparação entre a forma que o VE assume durante a sístole e o “tako-tsubo”, armadilha usada no Japão para capturar polvos. Em 2006, a American Heart Association classificou-a no grupo de cardiomiopatias adquiridas sob o nome de cardiomiopatia induzida por estresse.

As principais manifestações de Takotsubo são dor torácica, dispneia, alterações eletrocardiográficas de isquemia, discreto aumento de enzimas cardíacas e comprometimento segmentar da função ventricular, sem coronariopatia obstrutiva.^1^ Por apresentar quadro clínico semelhante ao das síndromes coronarianas agudas, o seu principal diagnóstico diferencial é com o infarto agudo do miocárdio (IAM), condição clínica de alta morbimortalidade, não havendo até o momento critérios que possibilitem estabelecer com clareza a distinção entre as duas patologias no momento inicial do atendimento ao paciente.

Estudos retrospectivos permitiram estabelecer as características prevalentes em indivíduos que apresentam takotsubo, como sexo feminino (90%), idade acima de 50 anos, histórico de estresse físico ou emocional recente, dor torácica de instalação aguda, supradesnivelamento do segmento ST (supra SST) ao eletrocardiograma (ECG) e aumento dos níveis séricos de troponina.,

A Sociedade Europeia de Cardiologia (ESC), em 2016, definiu os critérios diagnósticos para essa síndrome^1^ e estes foram os critérios utilizados no nosso registro. Posteriormente, em 2018, a ESC fez uma atualização nos seus critérios diagnósticos. Basicamente, as modificações foram a inclusão de feocromocitoma como causa específica de takotsubo e a possibilidade de coexistência de doença coronariana e takotsubo.

A fisiopatologia dessa síndrome é complexa e ainda não totalmente esclarecida. Diversos estudos apontam para liberação excessiva de hormônios adrenérgicos (epinefrina e norepinefrina) secundária a uma ativação simpática extrema e a resposta cardiovascular a essa ativação simpática súbita como os fatores centrais na fisiopatologia da doença.

As complicações decorrentes do takotsubo envolvem insuficiência cardíaca com frações de ejeção reduzida, geralmente abaixo de 25%, com hipocinesia apical (80%); insuficiência mitral moderada a grave (15% – 20%); choque cardiogênico (10% – 15%); morte intra-hospitalar (3% – 5%); e recorrência (5% – 10%).^4 , 5^ A evolução tende a ser benigna quando o suporte adequado é oferecido inicialmente, com reversão da disfunção ventricular entre uma e duas semanas, podendo levar até três meses.^4^

O estudo REMUTA é primeiro registro multicêntrico realizado no Brasil, envolvendo 12 centros privados no estado do Rio de Janeiro. Os objetivos da presente análise são descrever as características clínico-epidemiológicas de exames complementares, prognóstico e tratamento intra-hospitalar em pacientes diagnosticados com Takotsubo e também avaliar a mortalidade hospitalar e em 1 ano de seguimento.

## Métodos

### Definição de Subtipos Clínicos:

Primária: Os sintomas cardíacos agudos são a causa primária de busca de atendimento médico.

Secundária: Ocorre nos pacientes já hospitalizados por um motivo não-cardíaco. É uma complicação dessa condição primária ou do seu tratamento.

### Delineamento

Estudo observacional, de análise retrospectiva de prontuário médico. Para dados de mortalidade foram avaliados os registros do atestado de óbito do estado do Rio de Janeiro.

### Critérios de Inclusão e Exclusão

Foram incluídos os pacientes admitidos em hospitais privados com diagnóstico de takotsubo pelos critérios da ESC e aqueles que desenvolveram takotsubo durante internação por outra causa. Foram excluídos pacientes que apresentaram prontuário médico incompleto para os dados fundamentais de análise.

### Coleta de Dados

Foram coletadas as características clínicas, dados laboratoriais, radiografias de tórax, ecocardiogramas, dados eletrocardiográficos, ressonância nuclear magnética cardíaca e cateterismo cardíaco dos prontuários médicos. Cada coordenador de centro identificou os pacientes com takotsubo nos seus bancos de dados clínicos, no banco de dados do serviço de ecocardiografia ou do serviço de hemodinâmica. Depois da confirmação de que atendiam aos critérios de inclusão, foi preenchida uma ficha individual com os dados já descritos anteriormente. Dados de mortalidade foram coletados pelo banco de dados de óbito da secretaria de saúde do Estado do Rio de Janeiro.

### Objetivos

Descrever as características clínico-epidemiológicas de exames complementares, prognóstico e tratamento intra-hospitalar em pacientes diagnosticados com takotsubo. Avaliar a mortalidade hospitalar e em 1 ano de seguimento.

### Análise estatística

As variáveis contínuas foram descritas como média e desvio-padrão (DP) ou mediana e intervalo inter-quartil. Utilizamos teste t de Student não-pareado ou Mann-Whitney para comparação das variáveis contínuas e para identificar preditores univariados de mortalidade intra-hospitalar. Variáveis categóricas foram descritas como porcentagem. Foi empregado o Kolmogorov-Smirnov teste para testar o padrão de distribuição das variáveis numéricas. Utilizamos os testes exato de Fisher ou qui-quadrado para comparação das variáveis categóricas e para identificar preditores univariados de mortalidade intra-hospitalar. Variáveis que foram significantes na análise univariada foram incluídas numa análise multivariada (regressão logística) a fim de identificar preditores independentes de mortalidade. Valor de p < 0,05 foi considerado como tendo significância estatística.

Foram construídas curva de Kaplan-Meier para estimativa de sobrevida e comparadas através do teste de logrank. Análises uni- e multivariadas de Cox foram utilizadas para identificar preditores independentes de mortalidade após alta hospitalar.

O programa estatístico utilizado foi o SPSS versão 15.0.

### Aspectos éticos

O protocolo foi aprovado pelo Comitê de Ética em Pesquisa (CEP) da Casa de Saúde São José, RJ, em 26/11/2017, sob o número CAAE: 80206417.5.1001.5664 e número de parecer: 2.399.599.

## Resultados

Foram identificados 172 pacientes com critérios de inclusão. Após análise dos prontuários, 3 pacientes foram excluídos pois dados fundamentais para análise não estavam registrados nos prontuários. Portanto, foram incluídos na análise 169 pacientes, internados no período de outubro de 2010 a outubro de 2017, em 12 centros diferentes no Estado do Rio de Janeiro.

A idade média dos pacientes foi de 70,9 ± 14,1 anos e 90,5% do sexo feminino. Os sintomas prevalentes foram dor torácica (63,6%) e dispneia (44,6%). Histórico de estresse emocional esteve presente em 38,8% dos pacientes. A Tabela 1 mostra as variáveis clínicas da amostra estudada.

**Tabela 1:** – Variáveis clínicas da amostra

Variável	REMUTA (n = 169)
Idade (média ± DP)	70,9 ± 14,1
Sexo Masculino (%)	9,47
Dor torácica (%)	63,6
Dispneia (%)	44,6
Hipertensão Arterial (%)	69,7
Diabetes (%)	24,2
Dislipidemia (%)	37,6
Doença renal crônica (%)	5,4
FA/flutter (%)	21,2
Tabagismo (%)	17,6
Obesidade (%)	18,2
Estresse emocional (%)	38,8
PAS (mmHg) (média ± DP)	126,73 ± 25,2
PAD (mmHg) (média ± DP)	72,99 ± 15,6
PAM(mmHg) (média ± DP)	90,50 ± 17,8
FC (bpm) (média ± DP)	86,30 ± 20,0
Tempo de internação (dias) (mediana/IQR)	7,5 (5; 16)

*DP: desvio padrão; FA: fibrilação atrial; FC: frequência cardíaca; PAD: pressão arterial diastólica; PAM: pressão arterial média; PAS: pressão arterial sistólica.*

Na análise etiológica observamos que 63% dos casos foram de takotsubo primário e 37% de secundário.

Na admissão os pacientes se apresentavam com estabilidade clínica, refletida por pressão arterial sistólica (PAS) 126,73 ± 25,2 (média ± DP) e frequência cardíaca de 86,30 ± 20 (média ± DP).

Quanto aos exames complementares, troponina I foi positiva em 92,5% dos pacientes, com mediana (intervalo interquartil) de 2,37 (0,63;4,3) para convencional e de 24,3 (0,8;2650) para ultra-sensível. A mediana de BNP foi de 395 (176,5; 1725). Supra SST esteve presente em 28% dos pacientes, enquanto infradesnivelamento do segmento ST (infra SST) em 11,8%. A Tabela 2 mostra as principais características laboratoriais e de eletrocardiograma da população.

**Tabela 2:** – Variáveis laboratoriais e eletrocardiográficas

Variável (dados com registro/N total)	Resultado
Troponina positiva (161/169)(%)	92,5
CK-MB Positiva (84/169) (%)	84,7
Supra SST (161/169)(%)	28,0
Infra SST (161/169)(%)	11,8
BRE completo (161/169)(%)	7,1
Alteração repolarização (161/169)(%)	52,6
BNP (45/169) (pg/ml)(mediana/IQR)	395 (176,5; 1725)
Pró-BNP (7/169) (média ± DP)	4068,57 **±** 6121,28
Troponina I (45/169) (mediana/IQR)	2,37 (0,63; 4,3)
Troponina I US (76/169) (mediana/IQR)	24,3 (0,8; 2650)

*BRE: bloqueio do ramo esquerdo; infra SST: infradesnivelamento do segmento ST; supra SST: supradesnivelamento do segmento ST.*

Todos os pacientes fizeram coronariografia e em 24,2% doença coronariana não-obstrutiva (< 50%) esteve presente. Os outros 75,8% tinham artérias coronárias angiograficamente normais.

**Figura 1:**
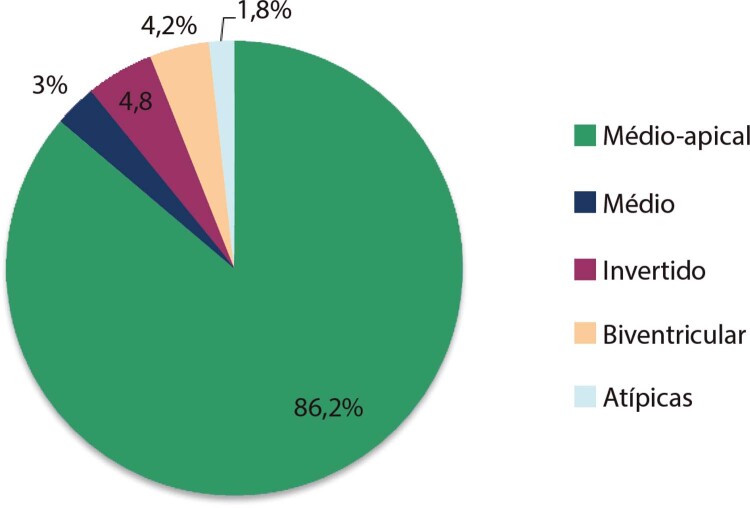
– *Padrão de alteração segmentar de contração.*

Quanto à análise ecocardiográfica, a fração de ejeção do ventrículo esquerdo (FEVE) teve mediana de 40 (35; 48)% quando avaliada pelo método de Simpson e de 48 (40; 62)% quando avaliada pelo método de Teichholz. Foi avaliada a presença de reversão completa ou parcial da disfunção do VE, e esta esteve presente em 68,2% dos casos. A Tabela 3 mostra as principais variáveis ecocardiográficas analisadas e a Figura 1 os padrões de alteração segmentar de contração.

**Tabela 3:** – Variáveis ecocardiográficas

Variável (n)	Resultado
FEVE Teichholz (143)(mediana/IQR)	48 (40; 62)
FEVE Simpson (87)(mediana/IQR)	40 (35; 48)
IM Moderada a grave (167) (%)	6,6
Trombo em VE ou VD (167) (%)	3,0
Derrame pericárdico (167) (%)	4,8
Obstrução do TSVE (166) (%)	4,2
Reversão da disfunção do VE (132) (%)	68,2

*FEVE: fração de ejeção do ventrículo esquerdo; IM: insuficiência mitral; TSVE: trato de saída do ventrículo esquerdo; VD: ventrículo direito; VE: ventrículo esquerdo.*

**Figura 2:**
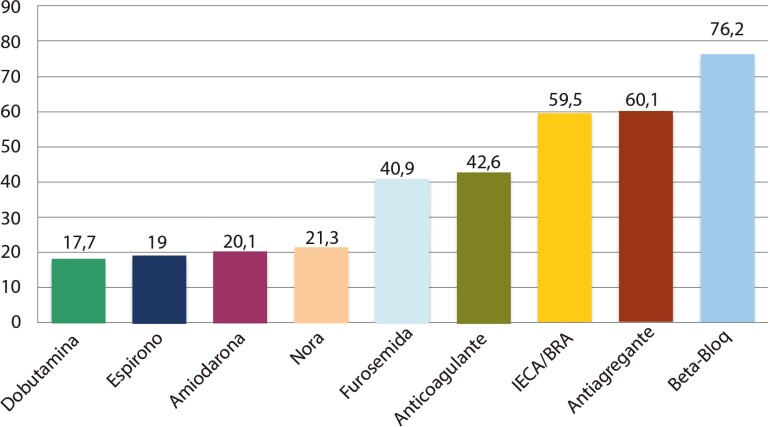
– *Fármacos utilizados durante a internação. Espirono: espironolactona; Nora: noradrenalina; IECA: inibidor do receptor da angiotensina; BRA: bloqueador do receptor da angiotensina.*

Quando analisamos os fármacos utilizados durante o período de internação, percebemos uma predominância de beta-bloqueadores (76,2%), antiagregantes plaquetários (60,1%), inibidores da enzima conversora de angiotensina ou bloqueadores dos receptores da angiotensina (59,5%), anticoagulantes (42,6%) e diurético de alça (40,9%). A utilização de dobutamina (17,7%) e noradrenalina (21,3%) também esteve presente numa parcela relativamente grande da população ( Figura 2 ).

**Figura 3:**
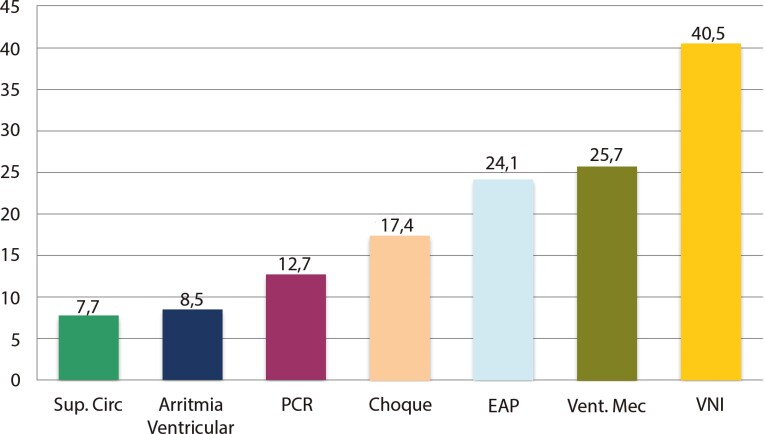
– *Complicações intra-hospitalares. PCR: Parada cardiorrespiratória; Sup. Circ: Suporte circulatório; EAP: Edema agudo de pulmão, VNI: ventilação não-invasiva; Vent. Mec ventilação mecânica invasiva.*

Quanto à evolução clínica intra-hospitalar, observamos que 40,5% dos pacientes necessitaram do uso de ventilação mecânica não-invasiva e 25,7% de ventilação mecânica invasiva. Edema agudo de pulmão foi observado em 24,1% dos pacientes e choque circulatório em 17,4%. Arritmia ventricular esteve presente em 8,5% dos pacientes, parada cardiorrespiratória em 12,7% e suporte circulatório mecânico foi utilizado em 7,7% dos casos ( Figura 3 ).

**Figura 4:**
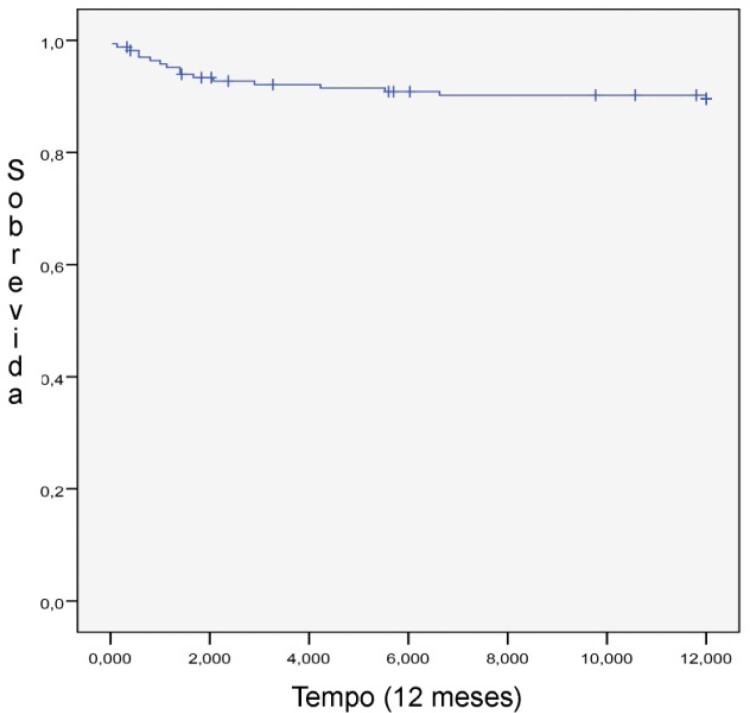
– *Sobrevida total em 1 ano.*

A mortalidade intra-hospitalar observada foi de 10,6% e a mortalidade ao final de 1 ano foi de 16,5% ( figura 4 ). Apenas 01 paciente com takotsubo primário evoluiu para óbito intra-hospitalar (0,91%), em comparação com 17 pacientes no grupo takotsubo secundário (28,3%). A Tabela 4 mostra a análise univariada de preditores clínicos e de exames complementares, com seu significado estatístico.

**Tabela 4:** – Análise univariada de preditores cínicos e de exames complementares

Variável	N	Vivos	Óbito	Valor de p
Idade (média ± DP)	169	70 ± 14	77 ± 14	0,056^δ^
Sexo				
Feminino	153	135	18	
Masculino	16	16	0	0,22*
Dor torácica				
Presente	105	100	5	
Ausente	60	48	12	0,002^#^
Dispneia				
Presente	74	63	11	
Ausente	92	86	6	0,078^#^
Troponina				
Positiva	149	134	15	
Negativa	12	11	1	1,0*
Supra SST				
Presente	45	41	4	
Ausente	116	105	11	1,0*
Infra SST				
Presente	20	17	3	
Ausente	140	128	12	0,4*
Estresse emocional				
Presente	64	63	1	
Ausente	101	85	16	0,003^#^
Takotsubo secundário				
Presente	60	44	16	
Ausente	105	104	1	< 0,0001^#^
Acometimento biventricular				
Presente	7	5	2	
Ausente	160	145	15	0,15*
Melhora da função ventricular				
Presente	90	83	7	
Ausente	42	34	8	0,77*
VNI				
Presente	68	55	13	
Ausente	100	95	5	0,004^#^
Arritmia ventricular				
Presente	14	10	4	
Ausente	151	138	13	0,041*
Ventilação mecânica invasiva				
Presente	43	28	15	
Ausente	124	121	3	< 0,0001*
PAM (mmHg) (média ± DP)	169	92 ± 17	81 ± 19	0,023^δ^
Choque cardiogênico				
Presente	29	19	10	
Ausente	138	130	8	< 0,0001*
Suporte circulatório mecânico				
Presente	13	8	5	
Ausente	155	142	13	0,006*

*teste exato de Fisher; #teste de qui-quadrado; δ Teste t de Student. DP: desvio-padrão; infra SST: infradesnivelamento do segmento ST; PAM: pressão arterial média; supra SST: supradesnivelamento do segmento ST; VNI: ventilação não-invasiva.

Para as variáveis com distribuição não-normal (troponina, BNP e fração de ejeção), utilizamos o teste de Mann-Whitney e apenas a fração de ejeção pelo método de Teichholz apresentou diferença significativa entre os grupos óbito e vivos (p = 0,001).

**Figura 5:**
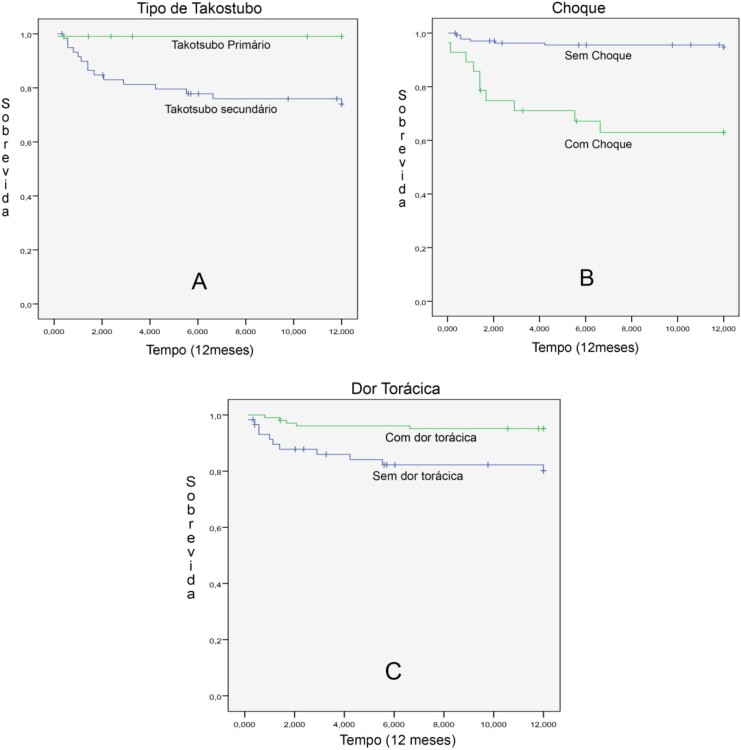
– *Preditores independentes de mortalidade. A: Sobrevida em 1 ano segundo tipo de takotsubo; B: Sobrevida em 1 ano segundo presença de choque; C: Sobrevida em 1 ano segundo presença de dor torácica na admissão.*

Analisando os preditores de óbito na análise multivariada (regressão logística *forward stepwise* ) observamos que takotsubo secundário (p = 0,035 e OR: 4,5) e choque cardiogênico (p < 0,001 e OR: 13,2) foram preditores independentes de mortalidade enquanto a presença de dor torácica foi fator protetor (p < 0,011 e OR: 0,14). A Tabela 5 mostra essa análise. A curva de sobrevida desses preditores se encontra na Figura 5 .

**Tabela 5:** – Análise multivariada dos preditores de óbito

Variável	B	Valor de P	OR	IC (95%)
Dor torácica	-1,99	0,011	0,14	0,03-0,6
Takotsubo secundário	1,5	0,035	4,5	1,1-18
Choque cardiogênico	2,6	0,001	13,2	3,0-59

*OR: odds ratio; B: constante de regressão; IC: intervalo de confiança.*

## Discussão

O presente estudo é o primeiro registro multicêntrico de takotsubo no Brasil. As informações mais importantes após análise dos dados foram: 1) A maioria das características clínico-epidemiológicas são semelhantes aos dos registros internacionais, isto é, predomínio de mulheres, idosas, com dor torácica e dispneia como sintomas prevalentes; 2) Estresse emocional foi encontrado em apenas 38% dos casos; 3) Observamos elevada taxa de takotsubo secundário; 4) A mortalidade intra-hospitalar foi elevada assim como ao final de 1 ano; 5) Takotsubo secundário e choque foram preditores independentes de mortalidade assim como dor torácica foi fator protetor.

Em relação a fatores desencadeantes, o estresse emocional não esteve presente na maioria dos pacientes. No registro Intertak, maior registro de takotsubo já publicado,^5^ essa taxa foi de 27,7% mostrando que a ausência de um fator emocional precedendo o quadro clínico não exclui em absoluto esse diagnóstico. E mais ainda, takotsubo precipitado por estresse físico geralmente tem causa secundária e pior prognóstico.

O BNP se mostrou elevado na nossa população. No Intertak^5^ o valor médio foi de 6 vezes o limite de corte para o teste. Esses valores foram maiores que os observados nos pacientes com síndrome coronariana aguda mas menores que o da população geral de insuficiência cardíaca descompensada, como no registro BREATHE que foi de 1075 (518; 1890).

Observamos uma prevalência de Supra SST menor que os 43,7% vistos no Intertak.^5^ Em contrapartida, nosso estudo teve uma taxa de Infra SST maior (7,7% no Intertak). Já um registro multicêntrico japonês de takotsubo mostrou elevada taxa de supra do SST, em torno de 74% e ondas T negativas em 70%. Esses dados nos mostram que alterações típicas de isquemia podem estar ausentes em 25% – 70% dos casos.

O grau de disfunção ventricular, refletida pela FEVE, foi igual àquele observado no Intertak^5^ (41% ± 11,8%). O padrão médio-apical foi, de longe, o mais encontrado, o que está em consonância com a literatura. Algo que chama à atenção no nosso registro é que o padrão biventricular veio em terceiro lugar. Isso não está bem descrito nos outros estudos de takotsubo, e mostra que devemos dar mais atenção à avaliação do VD nessa patologia. Complicações como insuficiência mitral moderada a grave, derrame pericárdico, trombo intra-ventricular e obstrução no trato de saída do VE foram observadas numa taxa não-desprezível, mostrando que o problema não é só a disfunção ventricular e que o acometimento cardíaco pode ser mais complexo em alguns casos. Outro ponto a destacar é o fato de que praticamente 1/3 dos pacientes pesquisados receberam alta sem experimentar melhora da função ventricular no ecocardiograma de controle pré-alta. Cabe ressaltar que apesar de, por definição, na síndrome de takotsubo a disfunção ventricular ser reversível, não existe tempo específico para essa melhora. Na nossa amostra a mediana do tempo de internação foi de 7,5 dias. Essa população deve ser acompanhada mais de perto no seguimento ambulatorial a fim de verificar se um tempo mais longo para recuperação da função ventricular tem algum impacto prognóstico.

O estudo Swedeheart, que avaliou 302 pacientes com takotsubo, mostrou choque cardiogênico em 5% dos casos e parada cardíaca em 3% enquanto no nosso registro essas taxas foram bem maiores. A utilização de inotrópicos e diuréticos foi 7% e 20%, respectivamente, no Swedeheart,^9^ o que está bem abaixo do observado no nosso registro, mostrando mais uma vez uma gravidade bem maior da nossa coorte. A mortalidade no Swedeheart^9^ foi de 4% em 30 dias, no registro japonês foi de 6,3% no período intra-hospitalar, no Intertak^5^ foi de 5,6% em 1 ano. Além da mortalidade significativamente maior no nosso estudo em comparação aos estudos internacionais, observamos ainda elevada taxa de complicações como choque, edema agudo de pulmão, necessidade de ventilação invasiva e não-invasiva e ainda 7,7% de utilização de suporte circulatório mecânico. Analisando os preditores de mortalidade no nosso registro identificamos que takotsubo secundário foi a grande responsável por essa elevada mortalidade. Esses pacientes que desenvolveram takotsubo no contexto de outra doença como causa da internação parecem ter características bem distintas daqueles com takotsubo primário. Artigo de revisão recentemente publicado mostra que no caso de takotsubo secundário a relação homem-mulher é bem mais equilibrada que na takotsubo primário, sendo de 1:1 a 1:3 no secundário e 1:9 no primário. Outra diferença importante é que presença de dor torácica no takotsubo primário é de 75% e no secundário é menor que 20%. Isso corrobora nosso achado de que dor torácica foi um fator protetor independente para mortalidade. Ainda, pacientes com takotsubo secundário tem maiores taxas de choque (30% – 69% x 9,9%) e de mortalidade intra-hospitalar (4,1% x 35% – 50%). São escassos na literatura dados de takotsubo secundário, mesmo dados simples como a incidência desse subgrupo nos registros. Uma revisão sistemática recentemente publicada, envolvendo 54 estudos observacionais com total de 4.679 pacientes com takotsubo avaliando prognóstico em longo prazo, mostrou mortalidade intra-hospitalar de 2,4%. A taxa anual de mortalidade no seguimento (mediana de 28 meses com intervalo interquartil de 23-34) foi de 3,5%. Na análise multivariada, foram identificados 3 preditores de mortalidade: idade mais avançada, forma atípica de balonamento ventricular e estresse físico. Isso corrobora nossos achados de que não se trata de patologia tão benigna e que estresse físico, que está intimamente ligado a takotsubo secundário, é fator prognóstico importante. As taxas de estresse físico nessa revisão sistemática^11^ e de takotsubo secundário no nosso estudo foram muito semelhantes: 36% e 37%, respectivamente. Ainda, nessa revisão sistemática a taxa de choque cardiogênico foi de 19%, bastante semelhante à do nosso estudo, e a taxa de arritmia maligna foi de 10%. Cabe ressaltar que essa revisão sistemática não incluiu nenhum estudo da América do Sul.

### Limitações

Este registro é uma análise retrospectiva de prontuário médico. Com isso, tivemos dados faltantes, especialmente de exames complementares, por ausência de seu registro no prontuário ou, mais provavelmente, por não terem sido realizados. Ressonância magnética (RM) foi realizada em apenas 20 pacientes (11,8%). Mas isso é uma característica comum nesse tipo de estudo e reflete a prática clínica. No registro japonês apenas 5,5% dos pacientes realizaram RM. Apesar do dado de mortalidade no seguimento de longo prazo ser bastante fiel, não temos dados de seguimento clínico pós-alta hospitalar.

## Conclusão

O REMUTA é o primeiro registro multicêntrico nacional de síndrome de Takotsubo. Seus resultados mostram não se tratar de patologia benigna como se pensava, especialmente no subgrupo de takotsubo secundário que carreia elevada taxa de complicações e de mortalidade. Estratégias de abordagem específica desse subgrupo devem ser desenvolvidas a fim de melhorar a qualidade do atendimento e os desfechos clínicos desses pacientes.
