# Supplementary material for: Registro Multicêntrico de Takotsubo (REMUTA) – Aspectos Clínicos, Desfechos Intra-Hospitalares e Mortalidade a Longo Prazo
Source: Arq Bras Cardiol. 2020 Aug 19;115(2):207–16. [Article in Portuguese] doi: 10.36660/abc.20190166 (PMC8384277; doi:10.36660/abc.20190166)
Supplement: Translation

# Translation: Takotsubo Multicenter Registry (REMUTA) – Clinical Aspects, In-Hospital Outcomes, and Long-Term Mortality

**Authors:** Gustavo Luiz Gouvêa de Almeida, João Mansur, Denilson Campos de Albuquerque, Sergio Salles Xavier, Álvaro Pontes, Elias Pimentel Gouvêa, Alexandre Bahia Barreiras Martins, Nágela S. V. Nunes, Lilian Vieira Carestiato, João Luiz Fernandes Petriz, Armando Márcio Gonçalves Santos, Bruno Santana Bandeira, Bárbara Elaine de Jesus Abufaiad, Luciana da Camara Pacheco, Maurício Sales de Oliveira, Paulo Eduardo Campana Ribeiro, Pedro Paulo Nogueres Sampaio, Gustavo Salgado Duque, Luiz Felipe Camillis, André Casarsa Marques, Francisco Carlos Lourenço, José Ricardo Palazzo, Cláudio Ramos da Costa, Bibiana Almeida da Silva, Cleverson Neves Zukowski, Romulo Ribeiro Garcia, Fernanda de Carvalho Zonis, Suzana Andressa Morais de Paula, Carolina Gravano Ferraz Ferrari, Bruno Soares da Silva Rangel, Roberto Muniz Ferreira, Bárbara Ferreira da Silva Mendes, Isabela Ribeiro Carvalho de Castro, Leonardo Giglio Gonçalves de Souza, Luiz Henrique dos Santos Araújo, Alexandre Giani

**Affiliations:** 1 Brazil Casa de Saúde São José, Rio de Janeiro, RJ - Brazil; 2 Brazil Hospital Samaritano, Rio de Janeiro, RJ - Brazil; 3 Brazil Universidade do Estado do Rio de Janeiro, Rio de Janeiro, RJ - Brazil; 4 Brazil Instituto D’or de pesquisa e ensino: ID’or/Cardiologia D’or, Rio de Janeiro, RJ - Brazil; 5 Brazil Instituto Nacional de Infectologia Evandro Chagas – FIOCRUZ, Rio de janeiro, RJ - Brazil; 6 Brazil Universidade Federal do Rio de Janeiro, Rio de Janeiro, RJ - Brazil; 7 Brazil Americas Medical City, Rio de Janeiro, RJ - Brazil; 8 Brazil Complexo Hospitalar de Niterói, Niterói, RJ - Brazil; 9 Brazil Hospital Universitário Antonio Pedro, Niterói, RJ - Brazil; 10 Brazil Clínica São Vicente, Rio de Janeiro, RJ - Brazil

**Keywords:** Cardiomyopathy, Dilated, Cardiomyopathy Takotsubo/mortality, Heart Failure, Stress, Psychological, Chest Pain, Dyspnea, Multicenter Study

## Abstract

**Background:**

Takotsubo syndrome (TTS) is an acquired form of cardiomyopathy. National Brazilian data on this condition are scarce. The Takotsubo Multicenter Registry (REMUTA) is the first to include multicenter data on this condition in Brazil.

**Objective:**

To describe the clinical characteristics, prognosis, in-hospital treatment, in-hospital mortality, and mortality during 1 year of follow-up.

**Methods:**

This is an observational, retrospective registry study including patients admitted to the hospital with diagnosis of TTS and patients admitted for other reasons who developed this condition. Evaluated outcomes included triggering factor, analysis of exams, use of medications, complications, in-hospital mortality, and mortality during 1 year of follow-up. A significance level of 5% was adopted.

**Results:**

The registry included 169 patients from 12 centers in the state of Rio de Janeiro, Brazil. Mean age was 70.9 ± 14.1 years, and 90.5% of patients were female; 63% of cases were primary TTS, and 37% were secondary. Troponin I was positive in 92.5% of patients, and median BNP was 395 (176.5; 1725). ST-segment elevation was present in 28% of patients. Median left ventricular ejection fraction was 40 (35; 48)%. We observed invasive mechanical ventilation in 25.7% of cases and shock in 17.4%. Mechanical circulatory support was used in 7.7%. In-hospital mortality was 10.6%, and mortality at 1 year of follow-up was 16.5%. Secondary TTS and cardiogenic shock were independent predictors of mortality.

**Conclusion:**

The results of the REMUTA show that TTS is not a benign pathology, as was once thought, especially regarding the secondary TTS group, which has a high rate of complications and mortality. (Arq Bras Cardiol. 2020; 115(2):207-216)

## Introduction

Takotsubo syndrome (TTS), also known as takotsubo cardiomyopathy or broken-heart syndrome, is a reversible regional dysfunction of the left (LV) and/or right ventricle (RV) in the absence of obstructive coronary disease; a large part of cases are caused by situations of acute stress. It was first described by Sato et al. in 1990, in Japan, with a series of 16 cases that presented clinical characteristics of acute coronary syndrome; all of the patients’ coronary arteries were, however, angiographically normal, and they had history of stressful event preceding the chest pain. The name is due to the similarity between the LV during systole and the “tako-tsubo,” which is a trap used in Japan for capturing octopus. In 2006, the American Heart Association classified it in the group of acquired cardiomyopathies, under the name stress-induced cardiomyopathy.

The main manifestations of TTS are chest pain, dyspnea, ischemic ECG changes, slight increase in cardiac enzymes, and impairment of segmental ventricular function, without obstructive coronary disease.^1^ Due to the fact that its clinical picture is similar to that of acute coronary syndromes, its main differential diagnosis is acute myocardial infarction (AMI), a clinical condition with high morbidity and mortality, and there are currently no criteria that make it possible to establish a clear distinction between the two pathologies during initial medical care.

Retrospective studies have made it possible to establish the most prevalent characteristics in individuals with TTS, such as female sex (90%), age over 50 years, recent history of physical or emotional stress, acute chest pain, ST-segment elevation (STE) on electrocardiogram (ECG), and increased serum levels of troponin.^,^

In 2016, the European Society of Cardiology (ESC) defined the diagnostic criteria for this syndrome,^1^ which were utilized in this registry. Subsequently, in 2018, the ESC updated their diagnostic criteria. Essentially, the modifications were the inclusion of pheochromocytoma as a specific cause of TTS and the possibility of coexisting coronary disease and TTS.

The physiopathology of this syndrome is complex, and it has not yet been fully made clear. Diverse studies indicate excessive release of adrenergic hormones (epinephrine and norepinephrine) secondary to extreme sympathetic activation and the cardiovascular response to this sudden sympathetic activation as the central factors in the physiopathology of the disease.

Complications that result from TTS involve heart failure with reduced ejection fraction, generally below 25%, with apical hypokinesia (80%), moderate to severe mitral regurgitation (15% – 20%), cardiogenic shock (10% – 15%), in-hospital mortality (3% – 5%), and recurrence (5% – 10%).^4 , 5^ Evolution tends to be benign when adequate support is provided early, with reversal of ventricular dysfunction in one to two weeks; reversal may, however, take up to three months.^4^

The REMUTA study is the first multicenter registry conducted in Brazil, involving 12 private centers in the state of Rio de Janeiro. The objectives of this study were to describe the clinical and epidemiological characteristics, complementary exams, prognosis, and in-hospital treatment of patients admitted with TTS and to evaluate in-hospital mortality and mortality during 1 year of follow-up.

(Veja comentário no texto original. A minha sugestão aqui seria “diagnosed”)

## Methods

### Definition of Clinical Subtypes

Primary: Acute cardiac symptoms are the main reason for seeking medical attention.

Secondary: This occurs in patients who were already diagnosed to the hospital for a different, non-cardiac reason; it is a complication of the primary condition or treatment thereof.

### Study Design

This is an observational study, with retrospective analysis of medical records. For data on mortality, death certificate records of the state of Rio de Janeiro were evaluated.

### Inclusion and Exclusion Criteria

Patients who were admitted to private hospitals with diagnosis of TTS according to the ESC criteria and those who developed TTS while already in the hospital for another reason were included in this study. Patients whose medical records were incomplete regarding data fundamental to analysis were excluded.

### Data Collection

Clinical characteristics, laboratory data, chest X-ray, echocardiogram, ECG, cardiac nuclear magnetic resonance, and cardiac catheterization were collected from medical records. Each center coordinator identified patients with TTS in their clinical databases or in the database of the echocardiography or hemodynamics service. Following confirmation that patients met the inclusion criteria, an individual form was filled out with the data previously mentioned. Mortality data was collected from the death database of the Secretary of Health of the state of Rio de Janeiro.

### Objectives

To describe the clinical and epidemiological characteristics, complementary exams, prognosis, and in-hospital treatment of patients diagnosed with TTS. To evaluate in-hospital mortality and mortality during one year of follow-up.

### Statistical Analysis

Continuous variables were described as mean and standard deviation (SD) or median and interquartile range. We used unpaired Student’s t-test or Mann-Whitney test to compare continuous variables and identify univariate predictors of in-hospital mortality. Categorical variables were described as percentages. The Kolmogorov-Smirnov test was used to test the distribution pattern of numerical variables. We used Fisher’s exact or chi-square tests to compare categorical variables and identify univariate predictors of in-hospital mortality. Variables that were significant in univariate analysis were included in multivariate analysis (logistic regression) in order to identify independent predictors of mortality. P-values < 0.05 were considered statistically significant.

Kaplan-Meier curves were constructed to estimate survival, and they were compared using the logrank test. Cox uni- and multivariate analyses were used to identify independent predictors of mortality after hospital discharge.

The statistical program used was SPSS version 15.0.

### Ethical Aspects

The study protocol was approved by the Research Ethics Committee of the Casa de Saúde São José, Rio de Janeiro, on November 26, 2017, under certificate (CAAE) number 80206417.5.1001.5664 and opinion number 2.399.599.

## Results

A total of 172 patients were identified with the inclusion criteria. After analysis of medical records, 3 patients were excluded, because data fundamental to analysis were not registered in the records. Therefore, analysis included 169 patients who were hospitalized between October 2010 and October 2017, in 12 different centers in the state of Rio de Janeiro.

Average patient age was 70.9 ± 14.1 years and 90.5% of patients were female. The most prevalent symptoms were chest pain (63.6%) and dyspnea (44.6%). History of emotional stress was present in 38.8% of patients. Table 1 shows the clinical variables of the study sample.

**Table 1:** – Clinical variables of the sample

Variable	REMUTA (N = 169)
Age (mean ± SD)	70.9 ± 14.1
Male sex (%)	9.47
Chest pain (%)	63.6
Dyspnea (%)	44.6
Arterial hypertension (%)	69.7
Diabetes (%)	24.2
Dyslipidemia (%)	37.6
Chronic renal disease (%)	5.4
AF/flutter (%)	21.2
Tobacco use (%)	17.6
Obesity (%)	18.2
Emotional stress (%)	38.8
SBP (mmHg) (average ± SD)	126.73 ± 25.2
DBP (mmHg) (average ± SD)	72.99 ± 15.6
MBP (mmHg) (average ± SD)	90.50 ± 17.8
HR (BPM) (average ± SD)	86.30 ± 20.0
Length of hospital stay (days) (median/IQR)	7.5 (5; 16)

*AF: atrial fibrillation; DBP: diastolic blood pressure; HR: heart rate; IQR: interquartile range; MBP: mean blood pressure; SBP: systolic blood pressure; SD: standard deviation.*

In etiological analysis, 63% of cases were primary TTS, and 37% were secondary.

Upon admission, patients presented with clinical stability, as reflected by systolic blood pressure (SBP) 126.73 ± 25.2 (average ± SD) and heart rate 86.30 ± 20 (average ± SD).

Regarding complementary exams, troponin I was positive in 92.5% of patients, with median (interquartile range) of 2.37 (0.63; 4.3) for conventional and 24.3 (0.8; 2650) for ultra-sensitive. Median BNP was 395 (176.5; 1725). STE was present in 28% of patients, while ST segment depression (STD) was present in 11.8%. Table 2 shows the population’s main laboratory and ECG characteristics.

**Table 2:** – Laboratory and electrocardiographic variables

Variable (recorded data/total N)	Result
Positive troponin (161/169)(%)	92.5
Positive CK-MB (84/169) (%)	84.7
STE (161/169)(%)	28.0
STD (161/169)(%)	11.8
Complete LBBB (161/169)(%)	7.1
Changes in repolarization (161/169)(%)	52.6
BNP (45/169) (pg/ml)(median/IQR)	395 (176.5; 1725)
Pro-BNP (7/169) (mean ± SD)	4068.57 ± 6121.28
Troponin I (45/169) (median/IQR)	2.37 (0.63; 4.3)
US Troponin I (76/169) (median/IQR)	24.3 (0.8; 2650)

*BNP: brain natriuretic peptide; LBBB: left bundle branch block; CK-MB: creatine kinase myocardial band; IQR: interquartile range; SD: standard deviation; STD: ST-segment depression; STE: ST-segment elevation; US: ultra-sensitive.*

All patients underwent coronarography, and non-obstructive coronary disease (< 50%) was present in 24.2% of cases. The other 75.8% had angiographically normal coronary arteries.

Regarding echocardiographic analysis, median left ventricular ejection fraction (LVEF) was 40 (35; 48)% when evaluated by the Simpson method and 48 (40; 62)% when evaluated by the Teichholz method. Complete or partial reversal of LV dysfunction was evaluated, and it was present in 68.2% of cases. Table 3 shows the main echocardiographic variables analyzed, and Figure 1 shows changes in segmental contraction patterns.

**Table 3:** – Echocardiographic variables

Variable (n)	Result
LVEF Teichholz (143) (median/IQR)	48 (40; 62)
LVEF Simpson (87) (median/IQR)	40 (35; 48)
Moderate to severe MR (167) (%)	6.6
LV or RV thrombus (167) (%)	3.0
Pericardial effusion (167) (%)	4.8
LVOT obstruction (166) (%)	4.2
Reversal of LV dysfunction (132) (%)	68.2

*LV: left ventricle; LVEF: left ventricular ejection fraction; LVOT: left ventricular outflow tract; MR: mitral regurgitation; RV: right ventricle*

**Figure 1:** – *Changes in segmental contraction patterns*

When analyzing medications used during the hospital stay period, we observed that betablockers (76.2%), antiplatelet agents (60.1%), angiotensin converting enzyme inhibitors or angiotensin receptor blockers (59.5%), anticoagulants (42.6%), and loop diuretics (40.9%) were predominant. Dobutamine (17.7%) and noradrenaline (21.3%) were also used in a relatively large portion of the population ( Figure 2 ).

**Figure 2:** – *Medications used during hospital stay. ACEI: angiotensin converting enzyme inhibitors; ARB: angiotensin receptor blockers*

Regarding in-hospital clinical evolution, we observed that 40.5% of patients required non-invasive mechanical ventilation, and 25.7% required invasive mechanical ventilation. Acute pulmonary edema was observed in 24.1% of patients, and circulatory shock was observed in 17.4%. Ventricular arrhythmia was present in 8.5% of patients; cardiorespiratory arrest was present in 12.7%, and mechanical circulatory support was used in 7.7% of cases ( Figure 3 ).

**Figure 3:** – *In-hospital complications. PCR: APE acute pulmonary edema; CRA: cardiorespiratory arrest; IMV: invasive mechanical ventilation; NIMV: non-invasive mechanical ventilation.*

In-hospital mortality was observed to be 10.6%, and mortality after 1 year was 16.5% ( Figure 4 ). Only 1 patient with primary TTS progressed to in-hospital death (0.91%), in contrast with 17 patients in the secondary TTS group (28.3%). Table 4 shows univariate analysis of clinical predictors and complementary exams with their statistical significance.

**Figure 4:** – *Overall 1-year survival.*

**Table 4:** – Univariate analysis of clinical predictors and complementary exams

Variable	N	Survival	Death	P value
Age (mean ± SD)	169	70±14	77±14	0.056^δ^
Sex				
Female	153	135	18	
Male	16	16	0	0.22*
Chest pain				
Present	105	100	5	
Absent	60	48	12	0.002^#^
Dyspnea				
Present	74	63	11	
Absent	92	86	6	0.078^#^
Troponin				
Positive	149	134	15	
Negative	12	11	1	1.0*
STE				
Present	45	41	4	
Absent	116	105	11	1.0*
STD				
Present	20	17	3	
Absent	140	128	12	0.4*
Emotional stress				
Present	64	63	1	
Absent	101	85	16	0.003^#^
Secondary takotsubo syndrome				
Present	60	44	16	
Absent	105	104	1	< 0.0001^#^
Biventricular involvement				
Present	7	5	2	
Absent	160	145	15	0.15*
Improved ventricular function				
Present	90	83	7	
Absent	42	34	8	0.77*
Non-invasive mechanical ventilation				
Present	68	55	13	
Absent	100	95	5	0.004^#^
Ventricular arrhythmia				
Present	14	10	4	
Absent	151	138	13	0.041*
Invasive mechanical ventilation				
Present	43	28	15	
Absent	124	121	3	<0.0001*
MBP (mmHg) (mean ± dp)	169	92±17	81±19	0.023^δ^
Cardiogenic shock				
Present	29	19	10	
Absent	138	130	8	<0.0001*
Mechanical circulatory support				
Present	13	8	5	
Absent	155	142	13	0.006*

*Exact Fisher test;^#^Chi-squared test;^δ^Student’s t test; MBP: mean blood pressure; SD: standard deviation; STD: ST-segment depression; STE: ST-segment elevation.

For the variables with non-normal distribution (troponin, BNP, and ejection fraction), we used the Mann-Whitney test, and only ejection fraction calculated by the Teichholz method showed significant different between the death and survival groups (p = 0.001).

In multivariate analysis of predictors of death (forward stepwise logistic regression), we observed that secondary TTS (p = 0.035 and OR: 4.5) and cardiogenic shock (p < 0.001 and OR: 13.2) were independent predictors of mortality, while the presence of chest pain was a protective factor (p < 0.011 and OR: 0.14). Table 5 shows this analysis. The survival curve of these predictors is shown in Figure 5 .

**Table 5:** – Multivariate analysis of predictors of death

Variable	B	P value	OR	CI (95%)
Chest pain	−1.99	0.011	0.14	0.03-0.6
Secondary takotsubo syndrome	1.5	0.035	4.5	1.1-18
Cardiogenic shock	2.6	0.001	13.2	3.0-59

*OR: odds ratio; B: regression constant; CI: confidence interval.*

**Figure 5:** – *Independent predictors of mortality. A: One-year survival according to type of takotsubo syndrome; B: One-year survival according to the presence of shock; C: One-year survival according to the presence of chest pain at admission*

## Discussion

This study is the first multicenter registry of TTS in Brazil. The most important observations after analysis of data were the following: 1) The majority of clinical and epidemiological characteristics are similar to those in international registries, namely, predominantly elderly women, with chest pain and dyspnea as the most prevalent symptoms; 2) Emotional stress was found in only 38% of cases; 3) We observed an elevated rate of secondary TTS; 4) In-hospital mortality was elevated, as was mortality after 1 year of follow-up, and 5) Secondary TTS and shock were independent predictors of mortality, while chest pain was a protective factor.

In relation to triggering factors, emotional stress was not present in the majority of patients. In the Intertak registry, the largest registry of TTS published to date,^5^ the rate was 27.7%, showing that the absence of an emotional factor preceding the clinical manifestation absolutely does not exclude this diagnosis. Furthermore, TTS preceded by physical stress generally has a secondary cause and a worse prognosis.

BNP was shown to be elevated in our population. In the Intertak registry,^5^ the average value was 6 times the cutoff limit for the test. These values are greater than those observed in patients with acute coronary syndrome but lower than those of the general population with decompensated heart failure, such as in the BREATHE registry where it was 1075 (518; 1890).

We observed a lower prevalence of STE than the 43.7% seen in the Intertak registry.^5^ On the other hand, out study had a higher rate of STD (7.7% in the Intertak registry). A multicenter Japanese registry of TTS showed an elevated rate of STE of approximately 74% and negative T waves in 70% of cases. These data show that typical ischemic changes may be absent in 25% to 70% of cases.

The degree of ventricular dysfunction, as reflected by LVEF, was equal to that observed in the Intertak registry^5^ (41% ± 11.8%). The mid-apical pattern was by far the one most found, which is in consonance with the literature. It is noteworthy that, in our registry, the biventricular pattern held third place. This is not well described in other studies on TTS, and it shows that we should pay more attention to the assessment of the RV in this pathology. Non-negligible rates of complications such as moderate to severe mitral regurgitation, pericardial effusion, intra-ventricular thrombus, and left ventricular outflow tract obstruction were observed, showing that ventricular dysfunction is not the only problem and that cardiac involvement may by more complex in some cases. Another point worth underscoring is that practically one third of our patients were discharged from the hospital without an improvement in ventricular function on pre-discharge control echocardiogram. It is worth underlining that, although, by definition, dysfunction ventricular is reversible in TTS, there is no specific time for this improvement. In our sample, median length of hospital stay was 7.5 days. This population should receive closer outpatient follow-up in order to verify if longer ventricular function recovery time has a prognostic impact.

The Swedeheart study, which evaluated 302 patients with TTS, found cardiogenic shock in 5% of cases and cardiac arrest in 3%, while these rates were much lower in our registry. The use of inotropic and diuretic medications was also 7% and 20%, respectively, in the Swedeheart study,^9^ which were much lower than the rates observed in our registry, showing, once again, the much greater severity in our cohort. Mortality in the Swedeheart study^9^ was 4% in 30 days; in the Japanese registry, it was 6.3% during the in-hospital period, and in the Intertak registry^5^ it was 5.6% over 1 year. In addition to the significantly higher mortality in our study, in comparison with international studies, we also observed a high rate of complications such as shock, acute pulmonary edema, need for invasive and non-invasive ventilation, and 7.7% use of mechanical circulatory support. Analyzing predictors of mortality in our registry, we found that secondary TTS was largely responsible for this high mortality. These patients who developed TTS within contexts where another disease was the reason for hospitalization appear to have very different characteristics from those with primary TTS. A recently published review article shows that, in secondary TTS, the men-women ratio is much more balanced than in primary TTS, namely 1:1 to 1:3 in secondary TTS, in comparison with 1:9 in primary TTS. Another important difference is that the presence of chest pain in primary TTS is 75%, while it is under 20% in TTS. This corroborates our finding that chest pain was an independent protective factor against mortality. Furthermore, patients with secondary TTS had higher rates of shock (30% – 69% versus 9.9%) and in-hospital mortality (4.1% versus 35% – 50%). In the literature, there is a scarcity of data on secondary TTS, for instance, even simple data on the incidence of this subgroup in registries. A recently published systematic review, involving 54 observational studies with a total of 4,679 patients with TTS evaluating long-term prognosis showed an in-hospital mortality of 2.4%. The yearly rate of mortality during follow-up (median of 28 months with interquartile range of 23 – 34) was 3.5%. Multivariate analysis identified the following 3 predictors of mortality: more advanced age, atypical form of ventricular ballooning, and physical stress. This corroborates our findings, namely, that this is not a benign pathology and that physical stress, which is closely linked to secondary TTS, is an important prognostic factor. The rate of physical stress in the systematic review^11^ and the rate of secondary TTS in our study were very similar, namely 36% and 37%, respectively. Furthermore, in the systematic review, the rate of cardiogenic shock was 19%, which is quite similar to our study, and the rate of malign arrhythmia was 10%. It is worth emphasizing that the systematic review did not include any studies from South America.

### Limitations

This registry is a retrospective analysis of medical records. For this reason, some data were missing, especially those from complementary exams, whether due to their absence from medical records or, more likely, because they were not performed. Only 20 patients (11.8%) underwent magnetic resonance (MR), but this is a common characteristic in this type of study, and it reflects clinical practice. In the Japanese registry, only 5.5% of patients underwent MR. Although mortality data during long-term follow-up tend to be quite reliable, we do not have data on post-discharge clinical follow-up.

## Conclusion

REMUTA is the first multicenter Brazilian registry of TTS. Its results show that TTS is not a benign pathology, as was once thought, especially in the secondary TTS subgroup which has an elevated rate of complications and mortality. Specific strategies for dealing with this subgroup should be developed with the aim of improving care quality and clinical outcomes for these patients.
